# A review of regenerative medicine and tissue engineering with a focus on wound healing and anti-aging

**DOI:** 10.3389/fsurg.2025.1504563

**Published:** 2025-06-05

**Authors:** Ovya Ganesan, Harriet Kiwanuka, Ryoko Hamaguchi, Dennis P. Orgill

**Affiliations:** ^1^ Geisel School of Medicine at Dartmouth, Hanover, NH, United States; ^2^ Plastic and Reconstructive Surgery, Brigham and Women’s Hospital, Boston, MA, United States; ^3^ Harvard Medical School, Boston, MA, United States

**Keywords:** tissue engineering, regenerative medicine, stem cells, aging, wound healing

## Abstract

Regenerative medicine and tissue engineering explore the potential to enhance human healing, which is often limited by wound contraction, scarring, loss of function, and decreased quality of life. Biomaterials like synthetic scaffolds and implantable devices have been developed to circumvent the body's limited natural ability to heal, however, they may introduce their own complications such as toxic side effects. Advances in cell-based therapies, especially those involving embryonic stem cells (ESCs) and human-induced pluripotent stem cells (hiPSCs), offer an enhanced ability to regenerate, circumventing limitations of biomaterials and the body's limited ability to heal. There have been many recent advances in cell-therapies, both scaffold-based and scaffold-free products. Additionally, non-cell-based therapies are gaining traction in wound healing. These products, utilizing their ability to affect the principles of wound healing, have applications in anti-aging. Despite these advances, significant challenges remain. These technologies remain costly, may compromise ethical tenets, and may introduce safety risks. Future work should address these challenges.

## Graphical Abstract

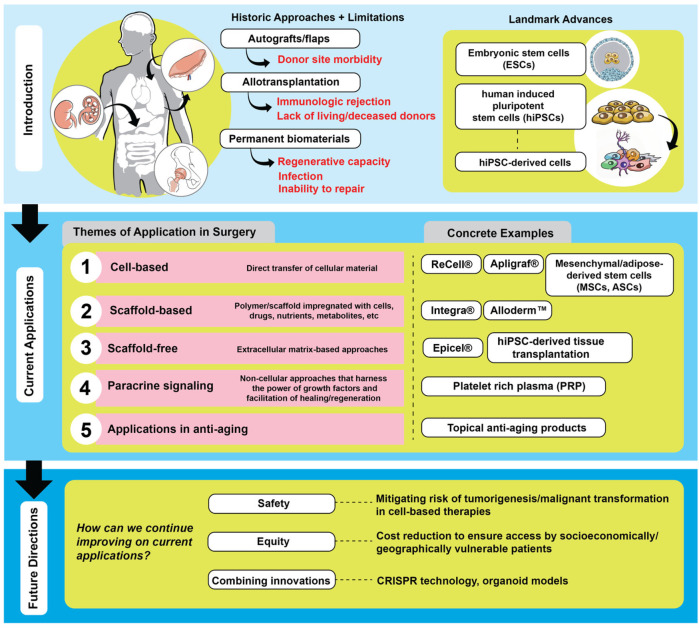

Visual abstract.

## Introduction

During human embryogenesis, multipotent stem cells differentiate into multiple cell lines and form a variety of complex anatomic structures. Unlike amphibians, which cam regenerate entire limbs after amputation, mammals have limited regenerative capabilities. Despite the conservation of DNA across species, factors beyond genetics may drive this dramatic difference in regenerative response.

Humans have a limited ability to regenerate but seem to respond to injury with an inflammatory response which results in wound contraction and scarring. Recent scientific advances have improved our understanding of the underlying principles of wound healing that lead to the outcomes that we see clinically. Application of this science has led to a wide array of products now used clinically to accelerate healing, reduce complications and improve long-term aesthetic results.

**Figure 1:**
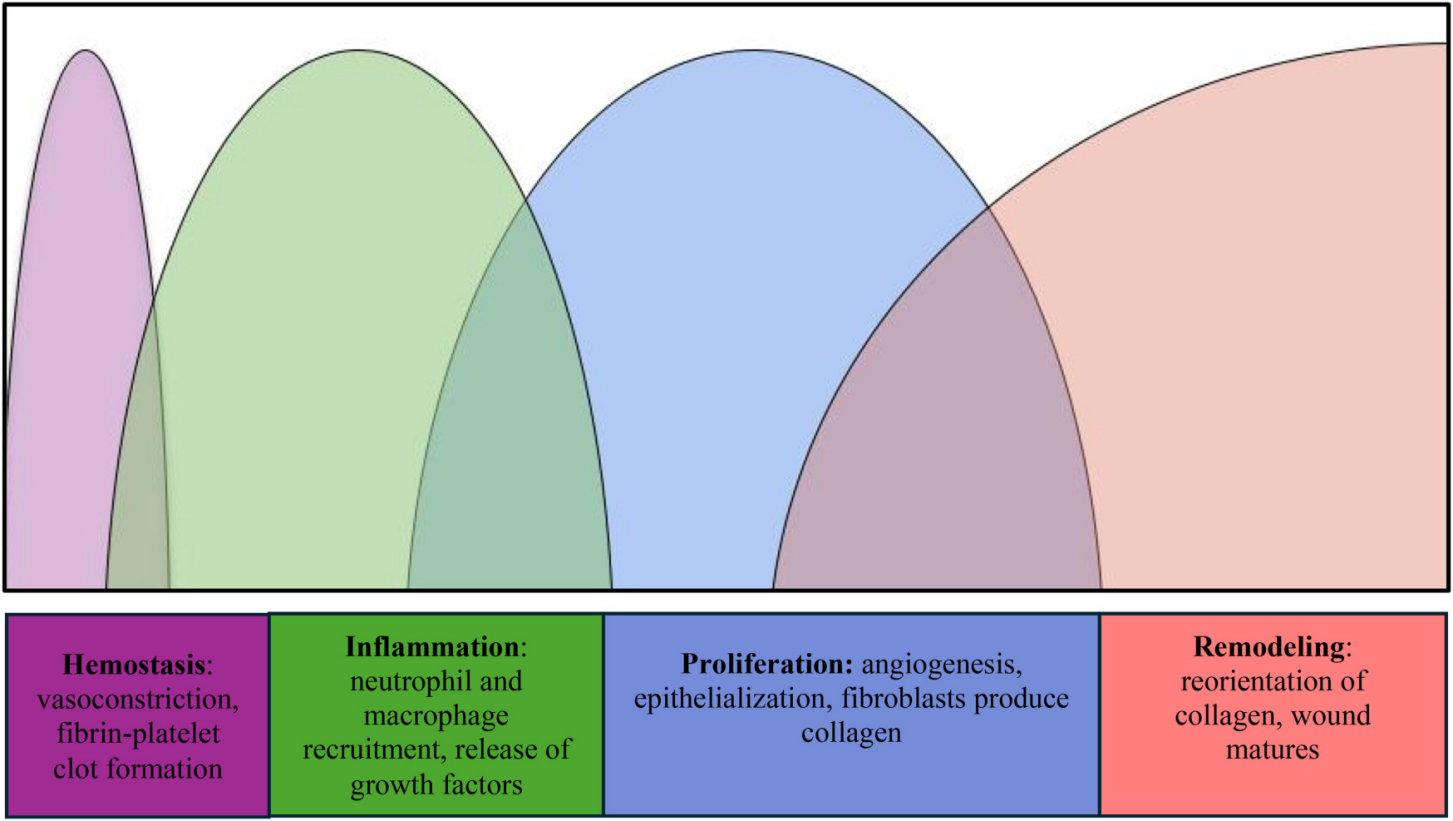
Depiction of the intensity of the phases of wound healing over time.

Researchers have worked on the assumption that a deeper understanding of salient cell types, cellular interactions, and proteins involved in wound healing will lead to interventions that can improve these processes. Wound healing is a complex phenomenon that encompasses the following distinct overlapping stages: hemostasis, inflammation, proliferation, and remodeling (Figure 1) (1). Hemostasis begins immediately after injury, driven by vascular constriction and the formation of a fibrin-platelet clot. Platelets play a key role by releasing vasoactive substances such as thromboxane A₂ and serotonin, which promote vasoconstriction, as well as growth factors like platelet-derived growth factor (PDGF) and transforming growth factor-beta (TGF-*β*), which initiate tissue repair. In the inflammatory phase, neutrophils clear pathogens and debris, while macrophages secrete cytokines and growth factors, including vascular endothelial growth factor (VEGF), to recruit additional immune cells and promote angiogenesis. During proliferation, fibroblasts synthesize collagen, endothelial cells drive new blood vessel formation, and keratinocytes support epithelialization of the wound bed. Finally, the remodeling phase involves myofibroblast-mediated contraction and matrix metalloproteinases (MMPs), which reorganize collagen fibers to increase tensile strength and restore tissue integrity (1, 2).

Despite these mechanisms, scarring and contractures often remain, limiting function and negatively affecting quality of life. True regeneration, defined as the replacement of new tissue without scarring, is a rare phenomenon in humans. For example, the regenerative capacity of cells of the central nervous system is transcriptionally turned off early in the developmental process, making it difficult for neuronal regeneration during adult life (3). The liver, while often cited for its regenerative ability, actually undergoes compensatory hyperplasia rather than true regeneration (3). The missing liver mass is replaced by the proliferation of existing hepatocytes without the activation of progenitor cells, contrary to actual regeneration (3).

Physicians have long leveraged the body's ability to adapt. Procedures such as local flaps, regional flaps, and free tissue transfer are routinely used in clinical practice to restore both function and aesthetics. These approaches have a proven track record with reliable outcomes. However, they do have their challenges. Flaps, grafts, and transplants contribute to sensory deprivation at the donor site, wound dehiscence, bleeding, infection, necrosis, scarring, and immune rejection (4–8). Despite immunological challenges, advances in immunosuppressive regimens and tolerance induction strategies have significantly improved outcomes. Recent work has suggested promising directions in immune modulation to prolong graft survival and reduce systemic toxicity (9). In cases of extensive tissue loss where conventional flaps are insufficient, vascularized composite allotransplantation is often the only viable strategy to restore both form and function. However, these approaches still fall short of restoring tissue to its original architecture and function.

**Figure 2:**
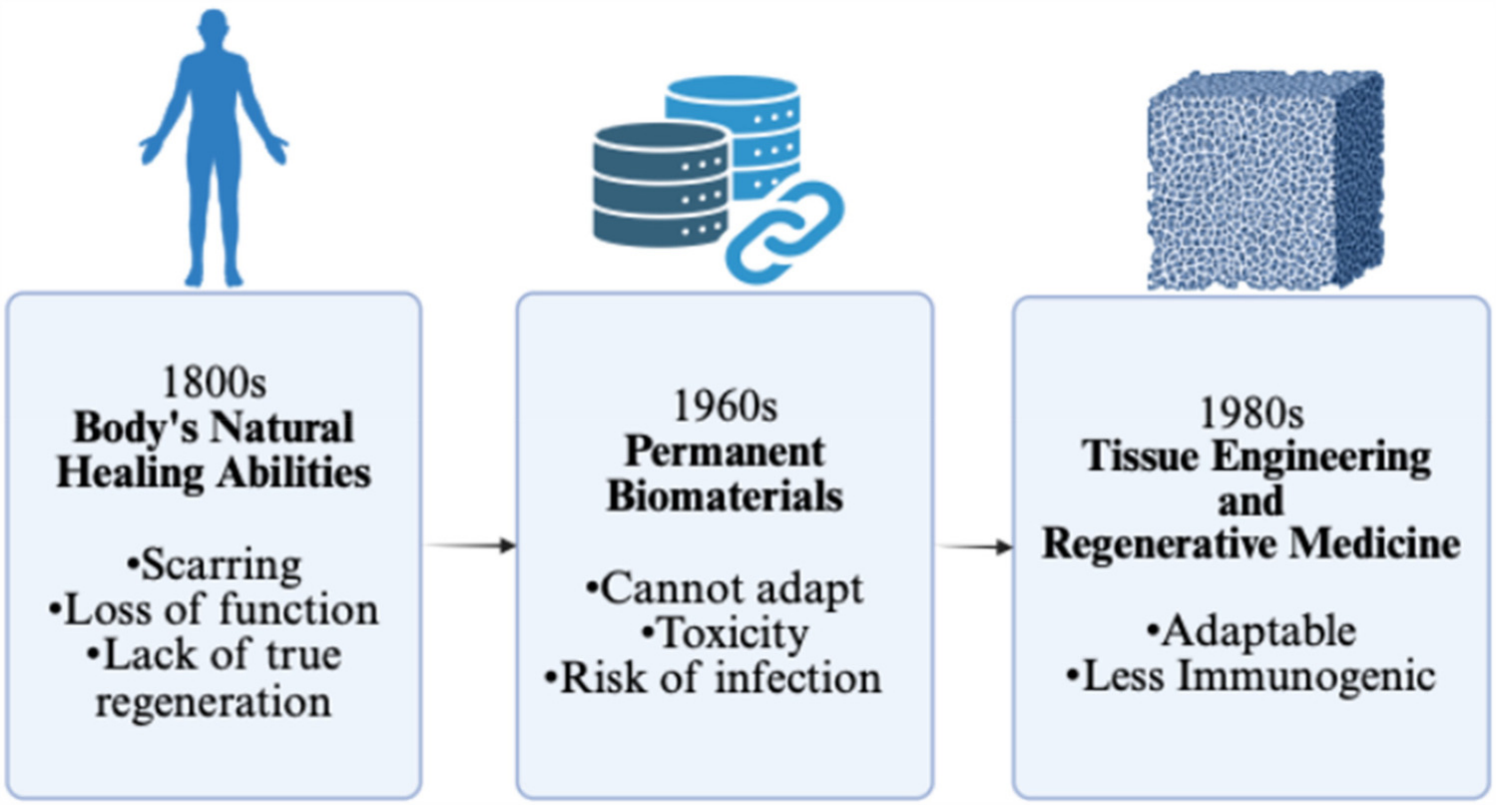
Schematic to demonstrate the progression of invention towards tissue engineering and regenerative medicine. Created with http://BioRender.com.

Given the body's limited innate regenerative capacity, scientists have explored biomaterials as a means of augmenting healing (Figure 2). These materials promote repair through several key mechanisms: they provide structural support to maintain tissue architecture, facilitate cellular infiltration, and serve as delivery platforms for growth factors, cytokines, and other bioactive molecules. Some are designed to mimic components of the extracellular matrix (ECM), enhancing cell adhesion, signaling, and tissue remodeling. Others are bioactive, stimulating angiogenesis or modulating the immune response.

Biomaterials may be categorized based on their purpose and permanence. Permanent implants, such as heart valves, joint replacements, and dental devices, are used to restore lost function. Temporary biomaterials, like dissolvable dressings, assist in wound coverage and tissue regrowth. Some also serve specialized roles, functioning as biosensors (e.g., glucose monitors) or drug delivery systems (e.g., coated vascular stents).

Despite these benefits, biomaterials often lack the dynamic responsiveness of living tissues. They cannot self-renew, remodel, or fully integrate with the host environment. Many serve more as passive frameworks than active participants in regeneration. This can result in immune rejection, foreign body reactions, fibrosis, or inadequate healing in complex wounds.

Additionally, biomaterials pose distinct challenges. They can be costly, symptomatic if they fail, and in some cases toxic. For example, chromium-cobalt hip implants have been linked to long-term complications such as metallosis, cognitive deficits, and musculoskeletal pain (10). Other risks include bleeding from anticoagulation required for mechanical heart valves or early deterioration of tissue valves (4). Even biologic solutions like flaps and grafts can result in donor site morbidity, infection, and scarring (6–8). Transplantation adds layers of risk from immunosuppression and immune dysfunction (9). Ultimately, none of these approaches possess intrinsic regenerative capacity, underscoring the need for more responsive and adaptive solutions (Figure 2).

## Overview of recent advents in regenerative medicine and tissue engineering

The limited regenerative capacity of both biomaterials and the adult human body has prompted extensive exploration of stem cell applications to regeneration. While the body's ability to regenerate is minimal in adulthood, it is most pronounced during early development. Embryonic stem cells (ESCs) found in the inner mass of a human blastocyst form the three normal embryonic layers during normal development. When extracted from the inner cell mass and cultured appropriately, ESCs retain their ability to proliferate indefinitely and differentiate into all three embryonic tissue layers *in vitro*. However, by definition, the extraction of ESCs implicates the sacrifice of the embryo itself.

The advent of human induced pluripotent stem cells (hiPSCs) has offered an exciting avenue to circumvent the potential ethical challenges associated with the use of ESCs (11). hiPSCs are derived from adult somatic cells that are reprogrammed to a pluripotent state and re-differentiated into a variety of cell types. This discovery, pioneered by Nobel Laureate Shinya Yamanaka and colleagues, found that hiPSCs can be generated by reprogramming adult somatic cells (such as dermal fibroblasts and peripheral blood mononuclear cells) into an embryonic stem cell-like pluripotent state through viral-mediated delivery of genetic factors such as octamer binding transcription factor 3/4 (*OCT3/*4), sex determining region Y-box 2 (*SOX2),* Kruppel-like factor 4 (*KLF4*) and cellular-Myelocytomatosis (*MYC)* (12). Since then, there have been ongoing efforts to increase the efficiency and yield of various reprogramming and differentiation protocols into a multitude of cell types (13–17). Research into applications in high throughput drug screening for toxicities and patient-specific disease modeling has been conducted for various pathologies including cardiac syndromes, neurodegenerative conditions, hematological disorders, and SARS-CoV-2 infection (18–23).

### Cell-based therapies

As scientists advance their manipulation of stem cells, cell-based therapies become more sophisticated. Cell therapy involves transferring regenerative cellular material into patients for therapeutic purposes (24). Examples include cord blood used for hematopoietic disorders, B-cell maturation agents used for large B-cell lymphoma, and autologous cellular products consisting of fibroblasts used for nasolabial fold wrinkles (24). Autologous therapies may deploy one cell type or leverage the synergy of multiple cells. For instance, Recell (ReCell® Avitas Medical Associates, Valencia, CA, USA) is an autologous suspension of keratinocytes, fibroblasts, and melanocytes that work in unison for both dermal and epidermal regrowth. Skin samples are obtained in the operating room and transferred to the laboratory for processing and preparation prior to reapplication (25).

Allogeneic therapies, such as Apligraf (Apligraf® Organogenesis, Canton, MA, USA) and Grafix (Grafix® Osiris Therapeutics, Inc., Columbia, MD), vary in the number of cell types included in its therapies (26). Apligraf is a bovine type I collagen matrix seeded with neonatal fibroblasts and keratinocytes (27). Over time, the fibroblasts produce a new dermis which is then overlaid by epidermal keratinocytes that eventually form stratified layers (27). Apligraf is known to contribute to faster healing and less fibrosis than natural skin (28). Placental constructs have been important in improving wound healing and decreasing inflammation and can come cryopreserved or dehydrated. Grafix, an example of a cryopreserved amniotic membrane containing mesenchymal stem cells, has improved outcomes for wound healing for diabetic and vascular patients (29).

Cell-based therapies also include the utilization of mesenchymal stem cells (MSCs) and adipose-derived stem cells (ASCs). MSCs are multipotent stem cells originating from bone marrow, and they release proangiogenic factors. They are more limited in their ability to differentiate compared to ESCs. However, multiple studies show the improvement of wound healing through the application of MSCs (29–32). ASCs are a subset of MSCs found in adipose. ASCs have promise in the field of tissue regeneration and wound healing as they are easily found in adipose tissue, differentiate into many lineages, and secrete a variety of cytokines (33). They have been used to reduce scarring in mice (34). Factors secreted by ASCs increased the rate of healing and reduced the deposition of collagen, allowing for finer and more organized tissues (34).

In recent years, there has been growing interest in the paracrine mechanisms of MSCs, particularly through MSC-derived exosomes (MSC-exos). These extracellular vesicles carry a variety of bioactive molecules including miRNAs, growth factors, cytokines, and lipids that play essential roles in angiogenesis, immune modulation, fibroblast activation, and extracellular matrix remodeling (35). Exosomal delivery of miR-21, miR-126, and other regulatory molecules has been shown to accelerate reepithelialization and improve vascularization in cutaneous wounds (36). Recent preclinical studies have demonstrated the utility of MSC-exos as a cell-free therapy. In murine and porcine wound models, MSC-exos enhanced reepithelialization, reduced inflammation, and promoted collagen maturation (37). In one study, exosomes derived from hypoxia-preconditioned MSCs further amplified these effects by increasing VEGFand TGF-β1 expression, suggesting a strategy for improving therapeutic potency (38).

Beyond wound care, MSC-exos have shown anti-aging effects, including reduction of oxidative stress, increased dermal thickness, and restoration of skin elasticity (39, 40). Their ability to promote mitochondrial function, reduce senescence markers, and increase collagen and elastin synthesis positions them as promising agents in regenerative dermatology and aesthetic medicine (39, 40).

MSC-exos offer a compelling advantage over traditional cell-based therapies: they mitigate risks of tumorigenicity, immune rejection, and ectopic tissue formation, making them a safer and more scalable alternative for clinical translation (38–41).

### Scaffold-based therapies

**Figure 3:**
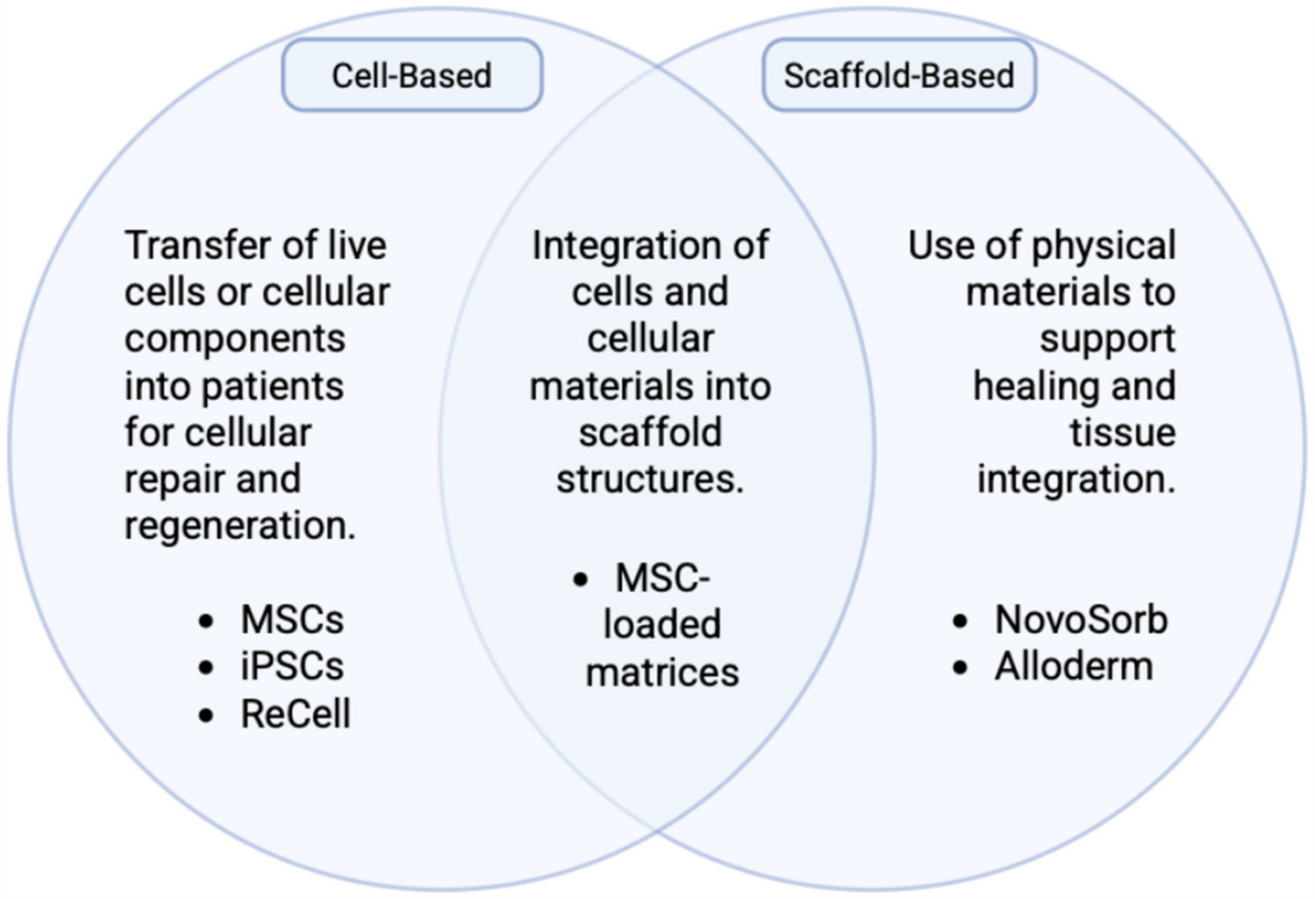
Schematic demonstrating the overlapping natures of cell-based therapies and scaffold-based therapies. Created with http://BioRender.com.

Cell-based therapies have expanded to include the addition of scaffolds, which have proven beneficial towards wound healing (Figure 3) (42). Scaffold-based therapies utilize a polymer or scaffold impregnated with cell types, drugs, nutrients, metabolites, or other substances to facilitate delivery or propagation within the body. Scaffolds can be synthetic, semisynthetic, decellularized, or composed of permanent and degradable materials (43).

The NovoSorb® Biodegradable Temporizing Matrix (BTM) (PolyNovo Biomaterials Pty Ltd., Port Melbourne, Australia) is a synthetic scaffold made of biodegradable polyurethane open-cell foam enclosed by a non-biodegradable membrane. This synthetic scaffold encourages vascular ingrowth, the development of new dermal tissue, and improved success rates of split-thickness skin grafts in burn victims (44). Integra (Integra® LifeSciences, Plainsboro, New Jersey), an acellular dermal matrix made of bovine tendon collagen, shark glycosaminoglycans, and silicone (45), is an example of a semisynthetic scaffold. With high take-rates, Integra matrices have helped improve wound healing in a plethora of clinical conditions, including lower extremity soft-tissue trauma, degloving injuries, hand wounds with exposed bone, and more (46). Decellularized scaffolds include Alloderm (Alloderm^™^ Allergan Aesthetics, Irvine California/ Dublin, Ireland), a dermal matrix made of human cadaveric collagen, lamin, and elastin (47), and Strattice (Allergan Aesthetics, Irvine California/ Dublin), a porcine-derived dermal matrix (48). Both have implications in reducing inflammation and fibrosis during the wound healing process (49).

### Scaffold-free products

There are also scaffold-free products that serve as tissue analogs and comprise an extracellular matrix. Epicel (Epicel® Vericel Corporation, Cambridge, MA, USA), a petroleum gauze made of sheets of autologous keratinocytes and proliferation-arrested murine fibroblasts, exemplifies a scaffold-free cell-based product that had been used for burn healing and cytokine and growth factor production (24).

Investigation into using hiPSCs in scaffold-free products has been robust. hiPSCs hold great promise for fabricating whole organs from patients' own cells, obviating the current limitations of immunogenic rejection and donor organ shortages. In 2017, Japanese researchers successfully transplanted a sheet of patient-specific hiPSC-derived retinal pigment epithelial cells into a patient with age-related macular degeneration, which remained intact at one year post-operatively but without significant improvement in visual acuity (50). The first in-human clinical trial of hiPSC-derived cardiomyocyte (hiPSC-CM) transplantation, published in 2023, reported three cases of hiPSC-CM patch constructs transplanted in patients with New York Heart Association Class III or higher heart failure. Results showed an improvement in heart failure symptoms and exercise tolerance as well as reduction in left ventricular diameter post-operatively (51). Finally, in the area of liver regeneration, researchers have successfully generated early fetal liver-like hiPSC-derived liver buds (52). These buds improved survival in murine subacute liver failure models (53).

Extending from this idea is that of the organoid, which is made of patient-derived stem cells grown in carefully selected media that determines differentiation then put onto an extracellular matrix to function as an organ (54). hiPSC-liver organoids have improved liver function and ameliorated chemically-induced liver fibrosis in murine models (55), suggesting that hiPSC-derived liver tissue may serve as a promising future therapeutic alternative to allogenic liver transplantation. Organoids have also been useful for drug screening, disease modeling, and tissue-specific functioning, successfully modeling lung and kidney function *in vitro* (24).

Recent studies have expanded the utility of organoids to model complex genetic disorders and to enhance their function through vascularization. In 2023, Bu et al. demonstrated that cerebral organoids derived from hiPSCs with *NANS* mutations accurately recapitulate human neurodevelopmental defects, offering insight into the mechanisms of cortical malformations and disease progression (56). In parallel, efforts to improve organoid viability and maturation have led to the development of kidney organoids co-cultured with human endothelial cells on microfluidic chips, resulting in vascular networks and enhanced organoid functionality (57).

Despite these advancements, ongoing challenges remain. One obstacle is that of precisely recapitulating the complex three-dimensional structure of native organs. Another concern is the risk of tumorigenicity and teratoma formation from proliferating progeny should hiPSCs or hiPSC-derived organs be transplanted into patients in the clinical setting.

### Paracrine signaling

Just as scaffold-free products promote cytokine and growth factor production, other cell-based therapies rely on the interplay between cells and their environments. Paracrine signaling is a crucial mechanism by which transplanted stem cells operate. Stem cells release paracrine factors that alter their extracellular matrix and ultimately influence their function. One example of this can be seen with post-infarction remodeling of heart chambers (58), where stem cells use paracrine signaling to enhance healing and reduce fibrosis. Researchers are exploring the isolation of these factors to develop therapies that could substitute stem cell treatments with soluble factor-based approached. If these factors can be effectively isolated, therapies may simply introduce them to enhance healing.

### Non-cell-based therapy

Platelet rich plasma (PRP) is made of the isolation of components of blood that consist of platelets, platelet-related growth factors, and plasma-derived fibrinogen. The platelets work differently from stem-cell based therapies in that they release growth factors for tissue repair rather than differentiate and divide to replace damaged tissue (59). PRP has been used for hair regrowth, tissue regeneration, wound healing, and musculoskeletal regeneration (60–63). In those with patellar tendinopathy, Achilles tendinopathy, and osteoarthritis, initial tests of injection of PRP have shown decreased inflammation, faster healing, and reduced the risk of tendon rupture (61, 63).

Given that PRP decreases inflammation, heals tissue damage, and reduces senescence, it naturally works against the accumulation of damage that leads to cellular aging (60). Injection of PRP has been used to stimulate ovulation in those with premature ovarian insufficiency and regenerate bone in osteoporosis and other conditions associated with aging (60, 64). However, there are inconsistencies within the literature regarding how to formulate the PRP for best use, and there is continued debate on the effectiveness of PRP (60). More robust prospective randomized clinical studies will be needed to better assess this therapy, and if the use of PRP can be perfected, the incidence of age-related disease may be able to be slowed.

Exosomes are another component that have promise in the realm of non-cell-based therapy. They are nanoparticles that hold molecules such as lipids, growth factors, and micro-RNA (mi-RNA). Different processes in the human body, such as cell development, growth, migration, and aging are mediated by particles released by exosomes (41). For example, specific mi-RNA in these exosomes may affect tissue regeneration and subsequently aging (41).

### Additional applications to aging

The science behind aging has become more advanced in recent decades, and patients have long been interested in how to avoid the effects of aging. Aging is often perceived as the cumulative result of cellular damage from environmental factors, leading to the natural possibility that the concepts of wound healing, which work to heal cellular injury, may be applicable to the aging process. In fact, young organisms, such as first and second trimester human fetuses, exhibit an ability to avoid scarring altogether. The mechanism of this process is unclear, but one hypothesis suggests intrinsic differences between fetal and adult skin rather than differences in the external environment (65). However, there are several hypotheses that argue for the role of the external environment's influence in aging. Epigenetic programming exemplifies this, and the “reprogramming” of a person's epigenetics has been studied as a means of reversing aging (66). Models based on DNA methylation levels have been shown to estimate the age of a person and predict risk of age-related disease with low precision, explaining 73% of age variance with a prediction error of 5.2 years (67). Another study hypothesized the benefit of heterochronic parabiosis, a procedure in which young and old mice are joined and share a circulatory system. The exposure of the old mouse to the young mouse improved the function of several organs and tissues in the old mouse (68). Studies have shown that circulating and systemic factors in young blood functionally improve aged brains, and counterpart factors in old blood contribute to aging phenotypes in younger tissues (68). If these factors are able to be isolated, patients might be able to benefit from a reduction in age-related chronic diseases.

**Figure 4:**
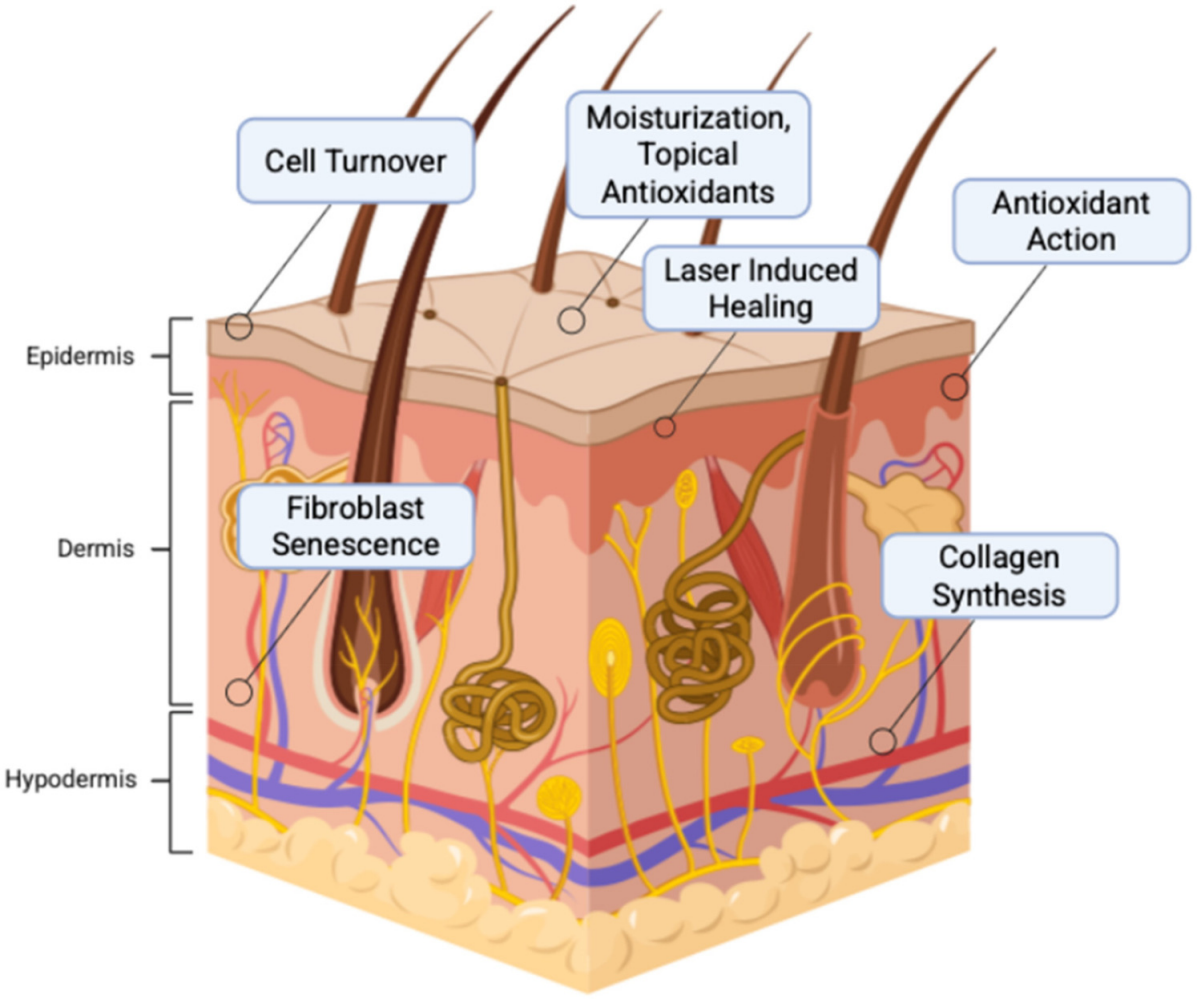
Mechanisms of anti-aging products that contribute to eliminating cellular mechanisms of aging. Created with http://BioRender.com.

Currently, popular, non-surgical anti-aging products include a range of topicals, metabolic supplements, and procedures. Topical products typically deliver antioxidants to neutralize free radical production from environmentally induced cell damage, improve moisturization and collagen production to restore the barrier between the environment and the skin, and increase cell turnover to remove pollutants that could contribute to cell injury (Figure 4) (69, 70). At the molecular level, antioxidants combat oxidative stress by neutralizing reactive oxygen species (ROS), which are byproducts of ultraviolet radiation, pollution, and metabolic processes that accelerate skin aging. Excessive ROS disrupt mitochondrial function, DNA integrity, and cellular membranes, leading to fibroblast senescence and impaired collagen synthesis (35, 71). Endogenous antioxidant systems including superoxide dismutase (SOD), catalase, and glutathione peroxidase work in tandem with exogenous antioxidants like vitamin C, vitamin E, and polyphenols to restore redox homeostasis (70, 72, 73). Activation of the Nrf2–ARE (antioxidant response element) signaling pathway is central to this process, triggering transcription of cytoprotective genes that reduce inflammation and support tissue repair (74). Furthermore, antioxidants enhance moisturization by stabilizing the skin barrier and boost collagen production by upregulating procollagen gene expression and inhibiting matrix metalloproteinases (MMPs), which degrade collagen during oxidative stress (75). These mechanisms collectively improve skin elasticity and hydration, key targets in regenerative anti-aging therapies.

Ultrasound technologies and laser therapies work by triggering a controlled wound-healing response so that collagen production, tissue remodeling, and cellular metabolism can be activated. Common supplements, like fish oil, lipoic acid, metformin, and nicotinamide mononucleotide work by reducing oxidative cellular injury.

Many surgical procedures performed to reduce signs of aging work to reverse visible damage at the macroscopic level rather than the cellular. Popular procedures performed on adult skin include brow or forehead lifts, blepharoplasty to reduce droopy eyelids, injectable facial fillers, and anti-wrinkle injectables such as Botox and laser skin resurfacing. Therefore, the utility of surgical procedures in anti-aging may be limited to cosmetic and physical alterations rather than targeting and preventing aging at a cellular level.

## Challenges and future directions

With any medical advancement, there are challenges that must be overcame for safe, ethical, and efficient delivery. One key concern is safety, particularly the potential for malignant transformation of cells used in cellular based therapies. Many of the same cell types and growth factors that play critical roles in wound healing are also important in tumor development. Relatedly, chronic wounds themselves carry an elevated risk of malignant transformation (76). Long term studies are needed to evaluate whether stem cell therapies increase the risk of malignancy in treated patients.

For chronic wounds, a multipronged approach remains standard. This includes debridement, moisture balance with advanced wound dressings, infection control, and offloading techniques in pressure-related ulcers. Adjuncts such as negative pressure wound therapy, bioengineered skin substitutes, and growth factor treatments are also commonly utilized (77). While regenerative approaches are under investigation, comprehensive wound care protocols remain essential in day-to-day management of chronic wounds.

The high cost of stem cell and bioengineered therapies may lead to violations of the ethical principles of justice and non-maleficence. These therapies may contribute to disparities in care due to their price. For example, a 2 × 2 inch piece of Integra can cost $2,500–3,000 dollars (78). As efficacy becomes better established, researchers must consider strategies for cost mitigation to support equitable access. Similarly, the sourcing of stem cells such as MSCs requires careful ethical consideration. Harvesting MSCs from long bones often involves bone marrow aspiration—procedures that, while minimally invasive, can cause pain and carry risk of complications. As a result, some investigators advocate for alternative sources such as adipose-derived stem cells (ASCs) or stem cells collected from discarded tissues, including liposuction specimens (79).

Paradoxically, the patients who stand to benefit the most from regenerative therapies are often those in whom these treatments are least effective. A common clinical scenario involves patients with severe vascular disease and poorly controlled type II diabetes who present with chronic lower extremity wounds. In such cases, bioengineered acellular scaffolds and other cell-based therapies may require multiple applications before sufficient revascularization and native cell ingrowth can occur. To improve the efficiency and cost-effectiveness of care, especially with expensive and resource-intensive therapies, future studies must evaluate these interventions specifically in clinically complex patient populations.

Looking ahead, several promising directions are emerging. Advancements in gene-editing technologies, such as clustered regularly interspaced short palindromic repeats (CRISPR), may allow for more precise stem-cell engineering, reducing the risks for tumorgenicity (80). More research into the optimal scaffold-based and scaffold-free platforms may enhance the delivery of cell-based therapies. Organoid models are being used to bridge the gap between *in vitro* models and clinical applications. Finally, increasing efficiency of biomanufacturing and reducing costs may help make therapies more accessible.
